# Conservation of oocyte development in germline cysts from *Drosophila* to mouse

**DOI:** 10.7554/eLife.83230

**Published:** 2022-11-29

**Authors:** Allan C Spradling, Wanbao Niu, Qi Yin, Madhulika Pathak, Bhawana Maurya

**Affiliations:** 1 Carnegie Institution for Science/Howard Hughes Medical Institute Baltimore United States; Whitehead Institute/MIT United States; University of California, Los Angeles United States

**Keywords:** oocyte, Balbiani body, germline cyst, nurse cell, developmental polarity

## Abstract

Recent studies show that pre-follicular mouse oogenesis takes place in germline cysts, highly conserved groups of oogonial cells connected by intercellular bridges that develop as nurse cells as well as an oocyte. Long studied in *Drosophila* and insect gametogenesis, female germline cysts acquire cytoskeletal polarity and traffic centrosomes and organelles between nurse cells and the oocyte to form the Balbiani body, a conserved marker of polarity. Mouse oocyte development and nurse cell dumping are supported by dynamic, cell-specific programs of germline gene expression. High levels of perinatal germ cell death in this species primarily result from programmed nurse cell turnover after transfer rather than defective oocyte production. The striking evolutionary conservation of early oogenesis mechanisms between distant animal groups strongly suggests that gametogenesis and early embryonic development in vertebrates and invertebrates share even more in common than currently believed.

## Introduction

The mature oocyte from which a child arises contains essentially all the molecules of the zygote, as the sperm provides almost nothing that lasts except a set of chromosomes and a centriole. This overwhelmingly maternal endowment includes not only rejuvenated cellular constituents but in many species vital developmental information that will initiate, pattern, and guide embryonic development. It has recently become clear that the oocyte-forming events that begin during the mother’s fetal development take place within a highly stereotyped cellular environment - the germline cyst - a multicellular structure that appears to have originated at the dawn of animal evolution, but which for years was studied mostly in *Drosophila* and other insects. How cysts support *Drosophila* and mammalian oocyte production by defining nurse cells (NCs) as well as oocytes, mediating organelle transfer, establishing the oocyte-specific organellar aggregate known as the Balbiani body (Bb) and possibly contributing to successful embryonic development will be reviewed here.

Studies of mammalian reproductive biology have well established the basic picture of how mammalian oocytes are formed and cyclically controlled (Edson et al., 2009; Richards and Pangas, 2010; Lesch and Page, 2012; Capel, 2017; Wu and Dean, 2020). Germ cells are initially specified by Bone Morphogenetic Protein (BMP) signaling during early gastrulation (Lawson et al., 1999; review Monsivais et al., 2017), after which they reprogram epigenetic marks (Sanford et al., 1987; Reik and Surani, 2015; Nicholls and Page, 2021; Saitou and Hayashi, 2021) and migrate to the gonads to initiate gamete production. A perplexing feature of mammalian oogenesis has been the large fraction of female germ cells that die rather than forming oocytes, especially during perinatal stages, suggesting that most germ cells are defective in some way and need to be culled (discussed in Tilly, 2001; Grive, 2020).

Studies of oogenesis in other animals, especially experimentally advantageous invertebrate models, allowed systematic analyses to genetically define genes important for gametogenesis (Nüsslein-Volhard, 2022; Schüpbach and Wieschaus, 1991; Hengartner and Horvitz, 1994; Dosch et al., 2004; Kimble and Crittenden, 2007; Strome and Lehmann, 2007). Widely conserved core genes (such as Vasa, Nanos, Piwi, Tudor, etc.) underlying the biology of all animal germ cells were identified (Fierro-Constaín et al., 2017; Sundby et al., 2021; Mercer et al., 2021) as well as hundreds of other maternally expressed genes, both conserved and more rapidly evolving, that contribute to oocyte production and early embryogenesis (Elis et al., 2018; Whittle and Extavour, 2019).

## *Drosophila* and invertebrate germline cysts

**Figure 1.**
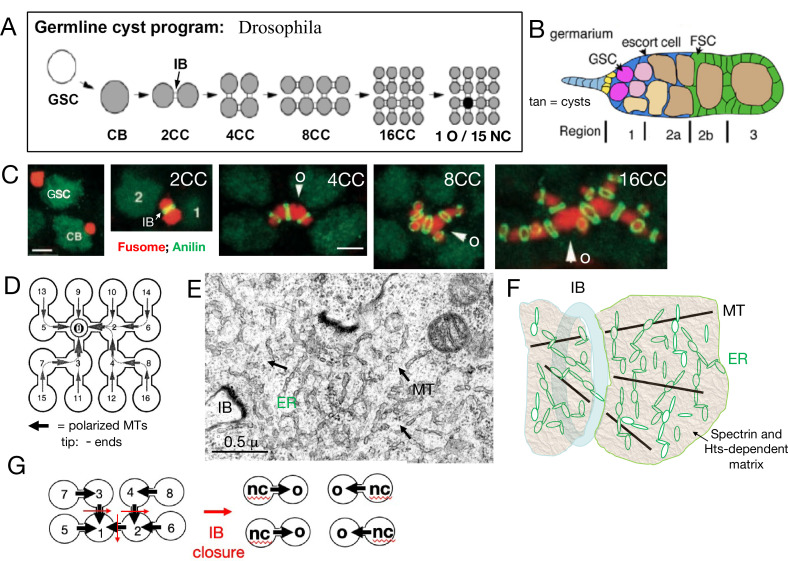
*Drosophila* germline cysts. (**A**) The germline cyst formation program. A germline stem cell (GSC) divides asymmetrically to generate a cystoblast (CB). The CB divides with incomplete cytokinesis to generate a two-cell cyst (2CC) with an intercellular bridge (IB). Three more cycles of rapid, synchronous, incomplete cyst divisions follow to generate 4CC, 8CC, and 16CC. One cell (dark) differentiates into an oocyte (O) while the other cells (gray) become nurse cells (NCs). (**B**) Germarium. A simplified *Drosophila* germarium is depicted showing regions 1, 2a, 2b, and 3. Shown are: GSCs (purple), forming cysts (pink), and 16CC cysts (tan). The somatic squamous escort cells (blue) surround all cysts in region 1 and 2a, while the follicle cells (green) generated by two follicle stem cells (FSC) displace escort cells and surround region 2b and later cysts. Follicle formation is completed in region 3. (**C**). Fusome asymmetry. The cyst fusome (red) spans cyst cells divided by IBs (green rings). Fusome asymmetry is evident after each division, with the first cell, "1" or arrowhead, destined to become the O, retaining the largest amount (de Cuevas and Spradling, 1998). (**D**) A 16CC cyst with arrows indicating the microtubule (MT) polarity (arrowhead tip = minus ends) derived from cyst divisions (cell numbers indicate production order). (**E**) Electron micrograph (EM) of the fusome near an IB. Arrows indicate MTs; ER-like vesicles are evident. (**F**) Fusome model showing an IB (blue), MTs (black), ER vesicles (green), and the alpha-spectrin and Hts-protein dependent matrix (tan). (**G**) The diagram shows how an 8CC with the indicated polarity can undergo fragmentation by closing (red arrows) IBs to yield four 2CC each producing one NC and O. Scale bars in C are 3μ (same for panels 1-2; or 3–5).

Developmental studies of the relatively simple and easily studied *Drosophila* ovary provided a cellular basis for understanding the genetic regulation of oogenesis (reviews: King, 1970; Mahowald and Kambysellis, 1980; Spradling, 1993). In particular, they highlighted the central role of a conserved cellular structure known as the germline cyst (reviews: Telfer, 1975; de Cuevas et al., 1997). Germline cysts are groups of germ cells interconnected by distinctive intercellular bridges (IBs) that are generated using synchronous mitotic divisions with incomplete cytokinesis (Figure 1A). In *Drosophila* and other insects, the IBs later acquire additional proteins and actin to support their expansion into large ‘ring canals’ (Robinson and Cooley, 1996). Cysts produced in this manner appear to be nearly universal in male gametogenesis and are found during early steps of oocyte production in diverse major animal groups including higher insects with complete metamorphosis where they persist longer and are most easily studied (Giardina, 1901; Wilson, 1925; Büning, 1994; Matova and Cooley, 2001; Lu et al., 2017). Many other invertebrates use interconnected syncytial cellular structures within their gonad for similar purposes that are built using a variant of the cyst formation program (Swiatek et al., 2009; Seidel et al., 2018; Świątek and Urbisz, 2019). However, cyst utilization has not been detected in females from some phylogenetic groups, a condition termed ‘panoistic.’ While in some panoistic species cyst formation early in development may have been overlooked, many such groups appear to have secondarily lost cyst production during evolution (Büning, 1994).

*Drosophila* oogenesis begins in the germarium (Figure 1B) at the tip of each ovariole that houses germline stem cells (GSCs). The first step downstream from a GSC is germline cyst formation. Cysts then participate in all the key developmental processes of early oogenesis including meiosis, oocyte specification, patterning, and Bb formation. Cysts influence these events by first acquiring an asymmetric cytoskeletal structure (Figure 1C–F) known as ‘the fusome’ (meaning ‘spindle derived’). This microtubule (MT)-dependent organelle grows with each successive mitotic division, interconnects all cyst cells, mediates germ cell cycle synchronization, and is essential for oocyte specification (Telfer, 1975; Lin et al., 1994). All cyst-forming divisions are asymmetric, with the older cell inheriting a larger amount of fusome than its daughter (Figure 1C; de Cuevas and Spradling, 1998). The central spindle MTs that initially arrest cytokinesis appear to be incorporated into the fusome in a polarized manner, allowing directed movement of cellular components toward the future oocyte to begin shortly after cyst completion (reviews: Spradling et al., 1997; Milas and Telley, 2022). Fusome MTs are stabilized by tubulin acetylation, the spectraplakin protein Shot, and the CAMSAP protein Patronin (Röper and Brown, 2004; Nashchekin et al., 2021). A simple and sufficient model is that fusome MT minus ends orient toward the older fusome segment as new segments attach following each cyst division cycle to generate a finished fusome-associated network capable of transporting cargos from any cyst cell toward the initial cell (Figure 1D).

The detailed molecular organization of fusome components remains incompletely understood, and some of the more than 20 identified fusome proteins change as cysts develop in the germarium (Lighthouse et al., 2008). Fusome-specific isoforms of several Adducin-like proteins produced by the *hu-li tai shao (hts*) gene (Petrella et al., 2007) and alpha-Spectrin are essential to prevent fusome dispersal, suggesting that these proteins play different roles in this non-membrane bound organelle than they do when associated with plasma membranes (de Cuevas et al., 1996). The presence of the Skp, Cullin, F-box containing (SCF) complex in the fusome controlling CycA and CycE turnover promotes the fusome’s ability to regulate germ cell cycles (Lilly et al., 2000; Ohlmeyer and Schüpbach, 2003). Finally, fusome-associated Endoplasmic reticulum (ER) proteins and vesicles provide ER connectivity between all cyst cells (Figure 1E; Snapp et al., 2004). A working model of fusome structure is shown (Figure 1F), in which its known components are proposed to be embedded in a Spectrin and Hts-fusome-dependent matrix (tan) that excludes ribosomes and most organelles, possibly due to phase separation.

Cysts carry out important roles during meiosis. Following four rounds of cyst-forming mitotic divisions, meiotic entry takes place, for which cyst cells have already been preparing their chromosomes (Christophorou et al., 2013; Rubin et al., 2016; Gyuricza et al., 2016). As meiotic prophase ensues, one cell is elaborated as the oocyte based on asymmetric fusome-mediated transfer from the other 15 cyst cells of RNAs, and likely other materials (see Hinnant et al., 2020). The transferring cells become a second cyst cell type known as nurse cells (NCs). NCs protect the oocyte by producing piRNAs (Guzzardo et al., 2013; Wang and Lin, 2021), promote oocyte growth and help to establish oocyte polarity and developmental patterning (review: Milas and Telley, 2022).

Somatic cells provide vital support to the developing germ cells throughout the germarium, often in response to germ cell Epidermal Growth Factor (EGF) and Notch signals. They communicate extensively among themselves and through signals to germ cells, along with humoral insulin, steroid and other hormonal signals, to coordinate all the early events of pre-follicular oogenesis with the nutritional environment (reviews: Laws and Drummond-Barbosa, 2017; Tu et al., 2021; Milas and Telley, 2022). Somatic cells also cause germline cysts to become oriented with respect to the anterior-posterior (a/p) axis of the ovariole starting in the germarium, which likely facilitates follicle formation and eventually ovulation (Godt and Tepass, 1998; González-Reyes and St Johnston, 1998).

Genetic studies strongly validate the importance of cysts and the fusome in specifying and producing oocytes. Mutations or treatments that disrupt cyst structure, the fusome, or MT-dependent transfer generate mostly NCs, fail to produce normal oocytes, and are female sterile (Koch and Spitzer, 1983; Yue and Spradling, 1992; Mach and Lehmann, 1997). Indeed production of a viable *Drosophila* oocyte without a cyst and NC transfer has never been documented (Schüpbach and Wieschaus, 1991; de Cuevas et al., 1997). In every species, the number of oocytes and NCs produced per cyst represents a fundamental parameter of female gamete development. Generally, a cyst of 2^n^ cells produces only 1 oocyte and 2^n^–1 NCs (‘the 2^n^ rule’). Production of more oocytes is usually associated with the fragmentation of the initial cyst into smaller cysts that then each follow the 2^n^ rule (Figure 1G; see Yamauchi and Yoshitake, 1982). How panoistic species are able to dispense with such an ingrained system while maintaining essentially the same sequence of developmental events remains an important issue.

## Mouse and vertebrate germline cysts

Evidence that developing mammalian male and female germ cells become connected by IBs was found beginning in the 1950s using electron microscopy (Burgos and Fawcett, 1955; review, Gondos, 1973), and similar findings were reported in *Xenopus* (al-Mukhtar and al-Mukhtar and Webb, 1971; Coggins, 1973). It was clear that the bridges were formed by incomplete cytokinesis and were only present during pre-follicular stages in females but persisted much longer in male gametes. A function in synchronization was proposed, but the presence of NCs was considered unlikely. Mouse primordial germ cells were eventually shown to undergo synchronized mitotic divisions to generate cell clusters up to 32 cells and preferentially containing 2^n^ cells (Figure 1C; Pepling and Spradling, 1998). The authors concluded that the cyst mechanism was conserved in mice based on comparisons of *Drosophila*, *Xenopus*, and mouse early germ cells (review: Pepling et al., 1999).

*Xenopus* oogonia were found to develop in 16 cell cysts (Kloc et al., 2004a) interconnected much like in *Drosophila*. However, due to the rarity of apoptotic germ cells, Kloc et al., 2004a concluded that all 16 cyst cells become oocytes. We now know that in mouse and *Drosophila*, NCs do not turn over primarily by apoptosis. Ovarian germline cysts containing up to 32 cells were subsequently discovered in the fish Medaka (Nakamura et al., 2010). Medaka ovaries contain ‘germinal cradles’ tipped with GSCs whose daughters immediately enter the cyst formation program, much like a *Drosophila* germarium. Medaka ovarian cysts form by 3–5 rounds of synchronous divisions, persist for a period to time, undergo some cell death, and produce oocytes in follicles (Nakamura et al., 2011). Similar cysts were also observed in zebrafish ovaries (Marlow and Mullins, 2008; Beer and Draper, 2013; Bertho et al., 2021).

**Figure 2.**
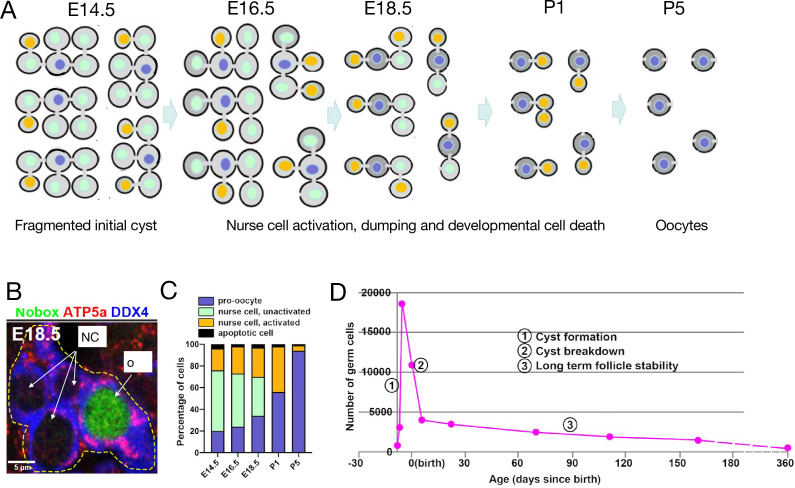
Mouse germline cysts. (**A**) A model of mouse primordial oocyte production from one initial 32cell cyst spaning the indicated ages (days, E=embryonic, and P=postnatal). Successive cohorts of unactivated nurse cells (green) beginning with outer cells, become activated (orange), begin to transfer cytoplasm and organelles causing them to shrink, and then turn over by PCD which occurs adjacent to the cyst and is not shown. From activation to Programmed cell death (PCD) requires about two days. (**B**) Immunostained image of a single E18.5 cyst (dashed yellow lines) showing DDX4 (germ cell marker, blue), ATP5a (mitochondrial marker, red), and Nobox protein (green). The oocyte (larger size and, mitochondria content), but not nurse cells, expresses Nobox (from Niu and Spradling, 2022). (**C**). Graph of mouse perinatal germ cell identity comprising nurse cells (unactivated, light green; activated, orange) and pro-oocytes (blue) as a function of developmental time. (**D**) Time course of mouse germ cell numbers per ovary vs time showing three phases. (1) sharp increase as primordial germ cells enter cyst formation; (2) sharp decrease due to cyst breakdown and programmed nurse cell death; and (3) very slow decline due to ovulation or atresia from the ovarian reserve (data from Lei and Spradling, 2013b; Lei and Spradling, 2013a).

The cellular products of individual cysts are very difficult to analyze by observation alone. Vertebrate ovaries may contain thousands of closely packed germ cells whose interconnections are nearly impossible to reconstruct even using confocal microscopy. Lei and Spradling, 2013a overcame this problem in mouse oogenesis by sparsely lineage marking individual migrating Primordial Germ Cells (PGCs) at E10.5, just as they were reaching the fetal ovary. This usually caused just one founder germ cell per ovary to be labeled, which produced one initial cyst of about 30 cells. Over the ensuing 10 days, about 4–6 labeled germ cells from the clone survived to become oocytes within 4–6 individual primordial follicles. Closer study showed that the initial cyst first fragmented into about 4–6 smaller derivative cysts by the time of meiotic onset, possibly in a programmed manner (Figure 2A). This suggested that each derivative cyst specifies one oocyte with the remaining cells functioning as NCs that turn over by the time primordial follicles finish forming around P5. Similar lineage marking will likely be necessary to characterize the fate of cyst cells in most species.

While the existence of vertebrate female germline cysts became clear, their functional importance remained controversial. Mutation of *Tex14*, encoding an important IB component and putative kinase known to function in cytokinesis, disrupted IB stability and caused male sterility, but females continued to produce fertile eggs in reduced numbers (Greenbaum et al., 2009; Greenbaum et al., 2011). A favored viewpoint remained that unlike *Drosophila*, female vertebrate cysts are not essential, lack NCs, do not transfer organelles but simply produce uniform oogonial cells that either die or became oocytes (Kloc et al., 2004a).

## Mouse ovarian cysts contain NCs that transfer organelles into oocytes

However, ongoing studies of the mouse showed that this view is incorrect. Cyst cells in each of the smaller cysts derived from a PGC, when lineage marked, could be seen to consist of NCs and an oocyte (Figure 2A). Organelles begin to move and accumulate in just one of the connected cells (Lei and Spradling, 2016). The transferring cells become reduced in size, while the receiving cell grows and gradually takes on the appearance of an oocyte as the transferred centrosomes and organelles build up in its cytoplasm. The distinct nature of NCs and oocytes was further revealed by their cell type-specific patterns of gene expression even while they remain connected (Figure 2B). After transfer, the NCs turn over, but they use a novel pathway of programmed cell death originally characterized in *Drosophila* termed ‘developmental cell death’ (Mondragon et al., 2019; Lebo and McCall, 2021) rather than apoptosis (Niu and Spradling, 2022). Thus, mouse cysts contain NCs and transfer most of their cytoplasm, centrosomes, and other organelles to an oocyte.

Further work demonstrated that the effects of *Tex14* mutation do not contradict the view that cysts play an essential role in mammalian oogenesis. Applying single germ cell lineage tracing to *Tex14* mutant ovaries showed that the fertility of Tex14^-/-^ animals is not due to oocyte production in the absence of cysts but because cyst production continues in the absence of stable IBs (Ikami et al., 2021). Without Tex14, cytokinesis still arrests, but due to IB instability, a reduced number of smaller cysts with a variety of structures are generated. Some *Tex14* mutant cysts become linked by membrane holes, like older normal cysts. These cysts still develop both cell types and NCs transfer cytoplasm to generate functional oocytes. Strikingly, the loss of Tex14 dramatically accelerates the timing of NC organelle transfer, suggesting that Tex14 controls the organelle transfer schedule (Ikami et al., 2021). Loss of Tex14 also alters and accelerates the onset of the normal spatial pattern of meiotic initiation (Soygur et al., 2021).

These insights provide a physiological explanation for the longstanding mystery of extensive germ cell loss during mouse fetal and early juvenile development (Tilly, 2001; Grive, 2020). Most germ cell organelles and cytoplasm are transferred to surviving oocytes prior to NC death (Figure 2C), so little biosynthetic investment and time are actually lost, while completed oocytes are highly stable (Figure 2D).

However, the structure of the initial mouse cyst and its pathways of processing to yield 4–6 oocytes still remain difficult to precisely reconstruct. Based on lineage marking, cyst intermediates appear to vary, suggesting that oocytes might develop from cysts of very different size. Yet relatively little variation is seen in the volume of oocytes in newly formed primordial follicles. It remains difficult to determine whether particular lineage-marked germ cells derived from a common progenitor retain a connection sufficient for cytoplasmic transfer, possibly even after moving some distance apart. Conversely, large clusters of adjacent marked sister germ cells may not all still be functionally connected. Because the number of transferred centrosomes observed in P1 oocytes and P5 oocyte size appears less variable than cyst interconnection patterns, we favor a model (Figure 2A) where cyst structure and breakdown are largely programmed by internal cyst asymmetries that arise during cyst formation and ensure structural similarity. However, individual PGCs do vary in the final number of oocytes produced (Lei and Spradling, 2013a), indicating that cyst breakdown is significantly less stereotyped in mouse than in *Drosophila*. Some reduced efficiency may have been an acceptable cost of increasing oocyte output per PGC by fragmenting the starting cyst.

## Differential oocyte and NC gene expression may control nursing

Careful study of lineage-marked sisters derived from one PGC revealed another surprising observation. Starting at E14.5, every 2 days 5–6 of the labeled PGC daughters not destined to become oocytes begin to transfer their cytoplasm, causing them to shrink into small cell remnants, before commencing programmed cell death (Figure 2A; Niu and Spradling, 2022). The first such NCs that become ‘activated’ for cytoplasmic transfer tend to be located at the edges of the cyst and have few connections to other cells. Later rounds move closer to the central cells with the most interconnections. Thus, mouse NC cytoplasmic dumping and cell death do not happen all at once, like in *Drosophila*, but are spread out over an approximately 10-day period. This behavior made it possible to identify stage-specific gene expression in activated NCs and oocytes using single-cell RNA sequencing (scRNAseq).

Immediately after cysts have formed by E14.5, both NCs and oocytes express largely the same genes characterized in previous studies analyzing total female germ cells. However, as the first NCs activate cytoplasmic transfer during leptotene/zygotene, their gene expression changes sufficiently to be separately characterized using scRNAseq. The ‘E14.5 NCs’ in these clusters contain strikingly less mRNA on average than the other leptotene/zygotene germ cells, confirming their NC identity. 2 days later, the leptotene/zygotene class of NCs has turned over, but a new group of pachytene NCs, ‘E16.5 NCs,’ with low average RNA content is observed whose gene expression differs from both the majority of pachytene cells and the earlier leptotene/zygotene NCs. Likewise, distinct gene expression is observed in dipolotene E18.5 NCs and in dictyate P1 NCs. Oocyte and unactivated NC gene expression coincides from E14.5 to E18.5, and beginning in P1 pure oocyte clusters can be sequenced.

Some highlights of NC and oocyte gene expression identified by scRNAseq are summarized in Table 1 (full data: see Niu and Spradling, 2022). The functional significance of the differentially expressed NC and oocyte genes has not yet been tested in cell type-specific knockout mice. However, some promising candidate genes that may contribute to germline cyst breakdown could be identified based on previous studies in whole animal mutants (Table 1).

**Table 1.** Oocyte and nurse cell (NC) gene expression.

Gene	Avg E14.5	E14.5 NC	E14.5 NC/Avg	Avg 16.5	E16.5 NC	E16.5 NC/Avg	Avg 18.5	E18.5 NC	E18.5 NC/Avg	P1 O	P1 NC	P1 NC/P1 O	P5 O
Oocyte													
Figla	30.60	10.30	*0.34*	47.19	45.56	*0.97*	147.10	132.35	*0.90*	204.59	94.24	*0.46*	63.14
**Nobox**	0.21	0.00	*0.00*	3.42	5.03	*1.47*	32.27	26.75	*0.83*	74.70	24.97	*0.33*	31.92
Padi6	0.21	0.00	*0.00*	0.00	0.00	*nd*	0.45	0.00	*0.00*	31.76	4.87	*0.15*	69.97
Filia/Khdc3	0.76	0.00	*0.00*	0.22	0.00	*0.00*	0.97	0.00	*0.00*	2.50	0.00	*0.00*	16.14
Floped/Ooep	11.64	8.32	*0.71*	3.22	0.00	*0.00*	16.40	12.11	*0.74*	57.59	25.07	*0.44*	80.86
Nlrp5/MATER	0.31	0.00	*0.00*	0.41	2.61	*6.37*	1.69	3.46	*2.05*	31.72	17.71	*0.56*	30.17
Tle6	5.16	4.26	*0.83*	6.27	0.00	*0.00*	28.24	18.47	*0.65*	75.58	32.94	*0.44*	59.98
E14.5 NC													
**Fst**	4.75	19.05	*4.01*	2.16	2.13	*0.99*	8.00	4.31	*0.54*	1.90	3.89	*2.05*	1.52
**Tex14**	79.30	24.30	*0.31*	58.70	15.20	*0.26*	34.10	18.70	*0.55*	26.00	3.10	*0.12*	9.44
**Pard6b**	3.34	10.51	*3.15*	4.15	0.00	*0.00*	4.22	0.89	*0.21*	9.72	0.33	*0.03*	1.81
Casp8	0.46	3.81	*8.29*	1.82	4.85	*2.67*	0.36	0.73	*2.00*	0.32	0.00	*0.00*	0.62
Wt1	4.04	12.60	*3.12*	3.74	4.05	*1.08*	6.58	10.79	*1.64*	5.18	3.01	*0.58*	2.42
Meis2	2.05	6.67	*3.24*	2.39	6.24	*2.61*	1.95	1.72	*0.88*	3.86	1.14	*0.30*	1.84
Kitl	3.49	14.92	*4.27*	6.17	13.98	*2.26*	10.05	5.95	*0.59*	5.03	0.00	*0.00*	0.09
Calb1	0.51	4.69	*9.17*	1.38	3.60	*2.60*	0.53	2.31	*4.34*	0.38	0.00	*0.00*	0.30
E16.5 NC													
**Fstl1**	6.51	7.71	*1.18*	4.00	12.00	*3.00*	3.77	2.07	*0.55*	4.69	3.20	*0.00*	1.71
**Cdkn1b**	4.08	11.85	*2.92*	5.96	13.47	*2.26*	2.70	0.00	*0.00*	1.94	0.00	*0.00*	7.91
Stmn3	0.60	4.05	*6.74*	0.20	1.66	*8.30*	0.15	1.30	*8.87*	0.00	0.00	*nd*	0.00
Jmjd8	1.34	4.84	*3.62*	0.81	2.61	*3.23*	3.20	3.46	*1.08*	2.61	1.38	*0.53*	1.60
Igfbp5	3.62	17.54	*4.84*	13.83	35.70	*2.58*	10.23	2.86	*0.28*	5.83	1.18	*0.20*	4.23
E18.5 NC													
Rspo1	2.28	2.13	*0.94*	0.49	2.61	*5.29*	1.16	2.65	*2.28*	2.56	1.34	*0.45*	0.13
Serpinb6b	0.51	2.25	*4.41*	0.57	1.04	*1.82*	0.60	2.18	*3.64*	0.55	0.00	*0.00*	0.41
P1 NC													
Cgn	3.80	6.17	*1.62*	1.59	2.75	*1.73*	2.01	0.00	*0.00*	0.99	3.10	*3.13*	1.98
Tmod1	0.74	7.32	*9.87*	2.67	0.00	*0.00*	1.30	0.00	*0.00*	0.66	1.97	*2.99*	0.14
Abhd2	5.13	9.23	*1.80*	1.06	2.23	*2.11*	1.72	0.72	*0.42*	0.18	2.37	*12.76*	0.58
Dusp3	0.13	3.28	*24.37*	0.33	1.64	*4.90*	0.71	0.00	*0.00*	0.60	2.29	*3.79*	1.04

Legend. Mouse ovary gene expression of the indicated genes at the indicate times in mixed cells (unactivated NC and oocytes, indicated by ‘Avg’), activated NCs or oocytes (O) were determined by single-cell RNA sequencing (scRNAseq; data from Niu and Spradling, 2022). Genes are grouped into those thought to function in oocytes (top section) and in lower sections, those thought to function in nurse cells peaking at the indicated times (i.e. E14.5 NC). Possible function of genes in **bold** are discussed in the text. E14.5=embryonic day 14.5, etc.; P1=postnatal day 1, etc.; ratios (NC/Avg) from the previous two columns are shown in italics. See text for discussion of specific genes. nd = not done.

Multiple genes are expressed specifically in newly forming oocytes and carry out functions important for oocyte development (review: Wu and Dean, 2020). Several tested genes in this class, including the key oocyte transcription factor *Nobox*, already express oocyte specifically (at the protein level) in cysts as early as E18.5 that still contain NC (Figure 2B, Table 1). In *Nobox*^-/-^ animals, cysts do not breakdown completely like in wild type (Rajkovic et al., 2004), suggesting that oocyte-specific *Nobox* expression within late cysts promotes normal breakdown, possibly by causing the oocyte to send transfer-promoting signals to its remaining NCs.

Changes in the expression of *Tex14* within activated NCs may also contribute to cytoplasmic transfer. *Tex14* expression levels in activated NCs fall three- to fourfold at E14.5 and E16.5 relative to pro-oocytes (Table 1). Reduced levels of Tex14 may accelerate transfer in the affected NCs, consistent with Tex14’s apparent role as a local transfer repressor (Ikami et al., 2021). Par family polarity protein mRNAs, including those encoding Pard3, Pard3b, Pard6a, Pard6b, and Mark3, are also downregulated in activated NCs (Pard6b, Table 1). Reduced Par expression may destabilize the NC cytoskeleton to facilitate cytoplasmic transfer into the oocyte.

Several genes upregulated in NCs may also promote cyst breakdown. The *Follistatin (Fst*) gene encodes an activin signaling regulator that is expressed about fourfold higher in activated E14.5 NCs than in pro-oocytes and unactivated NCs (Table 1). Fst exists in three isoforms, and whereas a global *Follistatin* deletion is embryonic-lethal, conditional ablation of all but the shortest isoform, FST288, identified essential roles of *Fst* in oogenesis (Kimura et al., 2011). In female FST288-only mice, cysts fail to breakdown on schedule, with breakdown prolonged by several days. Excess primordial follicles are generated that subsequently turn over rapidly, leading to premature ovarian failure. Interestingly, E16.5 NCs (Table 1) upregulate (3×) the level of a closely related gene, *Follistatin-like 1*.

Another gene promoting cyst breakdown is *p27Kip1 (Cdkn1b*) which is upregulated 2.9-fold in wild type E14.5 NCs compared to other E14.5 germ cells (Table 1). *p27Kip1* mutation increases the number of primordial follicles that are formed (Rajareddy et al., 2007) as well as the number of ‘multi-oocyte’ follicles (Pérez-Sanz et al., 2013); both phenotypes indicate that cysts in the mutant did not breakdown normally. Like in *Fst* mutants, in *p27Kip1* mutant animals, the excess and multi-oocyte follicles are defective, and few survive to produce offspring. An important caveat is that both *Fst* and *p27Kip1* are expressed at much higher levels in granulosa cells surrounding cysts than in their transferring NCs, indicating that these genes may promote cyst breakdown by acting in somatic cells.

## Why has oocyte development in cysts been highly conserved in evolution?

The studies reviewed here show that oocytes develop in germline cysts within species from diverse phylogenetic groups stretching back to the beginning of animal life more than 500 million years ago. Such conservation of a complex developmental pathway involving multiple cells of two cell types produced by a conserved stereotyped process is highly unusual and suggests that cysts serve an important purpose that has changed little over animal history. What might that purpose be?

One critically important role of the oocyte is to ensure that germ cells are fully restored in quality and do not retain damage that could progressively increase over multiple generations. Germ cells are well known to harbor systems that preserve their nuclear genomes (Bergero et al., 2021; Cao and Pocock, 2022). They also utilize mechanisms that select for and preserve undamaged mitochondrial DNA (Lieber et al., 2019; Chen et al., 2020; review Cox et al., 2021). Less is known about how damage to other cellular components is repaired. Studies in yeast show that meiosis is critically important for rejuvenation of cellular damage acquired during cellular aging. Of particular importance, during sporulation potentially damaging materials such as protein aggregates, aberrant nuclear pores, and excised rDNA circles are excluded from the four spore products, whose aging clock is concomitantly reset (Unal et al., 2011; Goodman et al., 2020).

Cysts are strong candidates to provide similar benefits to animal germlines, also in association with meiosis. Residual cytoplasm and organelles within NCs are destroyed by proteolysis and physically excluded from entering the oocyte. This provides an opportunity, at least in theory, for female germ cells to offload and purge themselves of materials each generation that have become too damaged or risky to repair. Thus, rather than simply promoting oocyte growth, oogenesis in cysts containing NCs may be a vitally important safety valve to prevent a multi-generational decline in oocyte quality. Such a decline might be underway even though the first post-cyst generation animals still appear grossly normal. Unlike somatic cells, which need only last one generation, germ cells must continue to function in perpetuity, which makes defects more difficult to test. It would not be surprising if germ cells housed unique structures and processes to support this major longevity challenge not seen in somatic cells.

A second reason that oocyte production in cysts may have been conserved relates to the oocyte’s preparations for embryonic development. Encoding and selectively utilizing genomic information to specify and produce a complex multicellular animal represent the most complex and sophisticated process known. Many vertebrate and invertebrate oocytes contribute not only rejuvenated cellular materials but also patterning information in the form of specified embryonic axes that guide early embryonic development. Evo-Devo comparisons of genomes, genes, and developmental pathways have revealed an underlying genetic similarity in how animal embryos, tissues, and their germ cells develop within different species, consistent with evidence that animals constitute a monophyletic group stretching back to a common animal ancestor (Fierro-Constaín et al., 2017; De Robertis and Tejeda-Muñoz, 2022).

It is plausible to consider that the germline cyst, the earliest conserved multicellular assembly on the developmental pathway of most major animal groups, contributes to the oocyte’s ability to launch embryonic development. Oocytes are currently believed to contribute less patterning information to mouse and mammalian embryonic development than do the oocytes of other well-studied animals such as *Drosophila* and lower vertebrates (Beddington and Robertson, 1999; Gardner and Davies, 2003; Innocenti et al., 2022), raising the question of whether patterning functions of cysts would be conserved in mammals.

## Cysts and centrosome migration to the oocyte contribute to Bb formation

**Figure 3.**
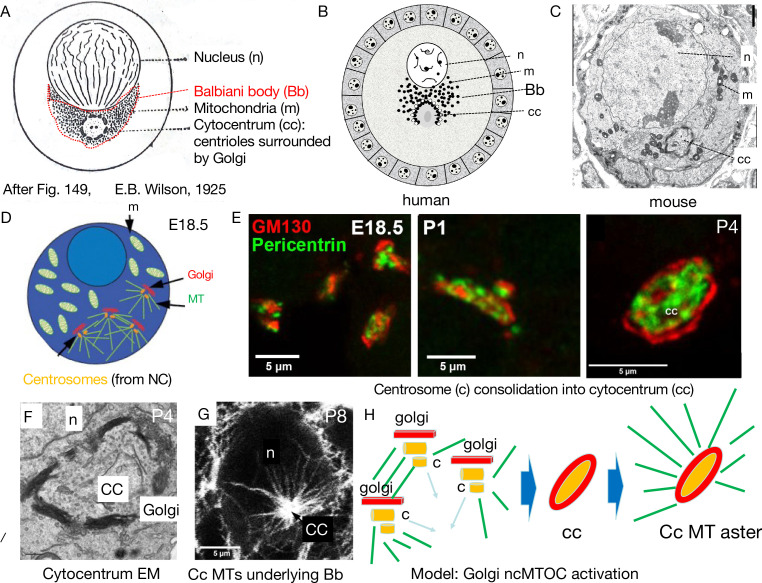
Mouse Balbiani body formation in a germline cyst from transferred nurse cell organelles. (**A**) Generic animal oocyte showing the nucleus (**n**), Balbiani body (**Bb**, red dashed border), mitochondria (**m**), and the cytocentrum (**cc**), consisting of centrioles surrounded by Golgi elements. Modified from Figure 149, Wilson, 1925. (**B**) Original drawing depicting a human primordial follicle showing the same structures as in (**A**) (see Hertig, 1968; Raven, 1961; van der Stricht, 1923). Magnification ×3000. (C) EM micrograph of a mouse P4 primordial follicle and Balbiani body showing prominent cytocentrum (cc). The mitochondria in mouse Bbs are less tightly clustered than in human Bbs. (**D–E**) The process of cytocentrum formation from transferred nurse cell centrosomes. (**D**) Diagram of an E18.5 oocyte showing four accumulated centrosomes transferred from nurse cells, each accompanied by a Golgi element. MT = microtubules. (**E**) The steps of cytocentrum formation. At E18.5, four centrosomes and accompanying Golgi are seen; at P1, centrosomes and Golgi have begun to coalesce; by P4, an finished cytocentrum (cc) has formed. Golgi (GM130, red) and Pericentrin (green). (**F**) EM of a P4 mouse cytocentrum. Scale bar = 2μ. (**G**) Immunofluoresence image of mouse P8 primordial follicle stained for alpha-tubulin (white), showing the massive microtubule aster nucleated by the cytocentrum (cc) that organizes the Balbiani body. n=oocyte nucleus; cc = cytocentrum. (**H**) Model of process depicted in D–G showing the switch from microtubule (MT) (green) nucleation by centrioles (c) to nucleation from the cytocentrum (cc) generated by centrosome coalescence and processing to yield an active Golgi-based non-centriolar MTOC (ncMTOC). (**D, E, and G**) are from Niu and Spradling, 2022.

If oocytes make a conserved developmental contribution, it is likely to involve early embryo asymmetry or patterning. An organelle known as the Balbiani body has long been of interest in developmental biology because its position in the early follicle relative to the oocyte nucleus (known as the germinal vesicle - GV) prefigures the animal/vegetal embryonic axis in diverse animal species including many vertebrates (Figure 3A; Wilson, 1925). A human Bb was observed more than 100 years ago as a large oocyte organelle cluster centered around a ‘cytocentrum’ in primordial follicles (Figure 3A and B; van der Stricht, 1923; Raven, 1961; Hertig, 1968; Kloc et al., 2004b). Similar Bb structures are found in mouse (Figure 3C; Pepling et al., 2007; Kloc et al., 2008), *Drosophila* (Mahowald and Strassheim, 1970), *Xenopus* (Kloc et al., 2004a), zebrafish (Elkouby et al., 2016), and even in panoistic insects (Tworzydlo et al., 2014).

The germline cyst contributes essentially to Bb formation in *Drosophila* (Cox and Spradling, 2003), mouse (Lei and Spradling, 2016), and probably many other species. Before the onset of meiosis or Bb appearance, the germline cyst’s polarity has been established, and at meiotic onset, this polarity mediates MT-dependent transport of materials that specify the oocyte(s) and begin to generate a Bb(s). During zygotene to dictyate meiotic stages, centrosomes and other organelles undergo MT-dependent transport from NCs to the oocyte (Cox and Spradling, 2003; Cox and Spradling, 2006; Lei and Spradling, 2016; Niu and Spradling, 2022). In mouse, the transported centrosomes coalesce in the oocyte and generate the cytocentrum (Figure 3D and E) consisting of pericentriolar material surrounded by Golgi membranes (Figure 3E and F). The cytocentrum, whose formation requires tubulin gene function (Niu and Spradling, 2022), nucleates a large MT aster (Figure 3G) on which oocyte organelles associate to form the Bb (Lei and Spradling, 2016; Niu and Spradling, 2022).

Thus, MTs play critical roles in Bb production by transporting organelles to the oocyte from NCs, forming the cytocentrum, and organizing oocyte organelles into a characteristic Bb structure on nucleated MTs. MT disruption at any stage in this process abolishes Bb formation. In *Drosophila*, the number of mitochondria in the Bb can even be manipulated over a wide range by mutating MT motors or the two Milton mitochondrial transport adaptor protein isoforms expressed in oocytes (Cox and Spradling, 2006).

NC centriole migration to the oocyte prior to follicle formation was originally described in *Drosophila* (Mahowald and Strassheim, 1970). NC centrioles move along the fusome and arrive at the oocyte as the follicle begins to form at the onset of stage 1 then rapidly move to the posterior where they aggregate as revealed by EM (Mahowald and Strassheim, 1970) and by gamma-tubulin staining (Grieder et al., 2000). The aggregated centrioles may represent a *Drosophila* analog of the cytocentrum. As stage 1 continues, the posterior MTOC forms (Theurkauf et al., 1993) and the Bb coalesces (Cox and Spradling, 2003).

The functional role of centriole migration in *Drosophila* for generating the posterior MTOC has remained uncertain, and this MTOC is likely non-centriolar MTOC (ncMTOC; Bolívar et al., 2001; Nashchekin et al., 2021). Moreover, disruption of the centriolar replication factor SAS-4 eliminates centrioles but does not block oogenesis (Stevens et al., 2007), possibly because peri-centriolar material sufficient for posterior MTOC formation is still available in the oocyte (Zhao et al., 2012). Centriole migration may have other functions as well (Braun et al., 2021). Centriole migration was blocked by targeted alteration of the APC/C-specific E2 ligase Vihar, which caused premature polo kinase degradation and centriole destabilization in the oocyte without altering the fusome. Without centriole migration, the oocyte still formed but could not be maintained due to ectopic MT nucleation in NCs that probably interfered with NC to oocyte transport. Thus, relocating NC centrioles to the oocyte may help establish and maintain a stable MT cytoskeleton in both cell types.

## The cytocentrum may activate ncMTOC activity in Bb-associated Golgi

The fact that centrosome/centriole migration has been conserved between *Drosophila* and mouse argues strongly that this process serves an important function. We suggest that these migrations act as a switch to activate new ncMTOC formation that organizes the Bb and defines the Bb-GV axis that presages the *Drosophila* a/p axis. In mouse oocytes, centrioles are not observed in the finished mouse cytocentrum by EM, which contains a pericentrin-rich core and is flanked by prominent stacked Golgi membranes that define its edges (Figure 3E and F). Based on its size and shape, we propose that the mouse cytocentrum activates a powerful ncMTOC on cytocentrum-associated Golgi membranes (Figure 3G and H). Likewise, *Drosophila* centriole migration may activate ncMTOC activity at the oocyte posterior, where Golgi elements cluster (Cox and Spradling, 2003).

A variety of cells and fibroblast cell lines in addition to a centriolar MTOC have a second, Golgi-associated ncMTOC (Rios, 2014; Valenzuela et al., 2020). Golgi-nucleated MTOCs utilize Cdk5rap2, which binds to the gamma-tubulin ring complex, as well as tubulin polymerization promoting protein (TPPP) family members. Both Cdk5rap2 and TPPP3 are expressed in dictyate mouse oocytes based on scRNAseq. Additionally, CAMSAP proteins bind and stabilize Golgi-nucleated MT minus ends, including the mouse oocyte-expressed CAMSAP1, a Patronin ortholog. In cultured cells, loss of the centriolar MTOC can cause Golgi-dependent MT nucleation to become predominant (Gavilan et al., 2018; Martin and Akhmanova, 2018; Chen et al., 2022).

## The Bb is important for oocyte axis specification

Studies in *Xenopus* (Kloc et al., 2004b; King et al., 2005) and zebrafish (Jamieson-Lucy and Mullins, 2019) showed that germ granules and mRNAs become localized within the oocyte Bb throughout stage 1 after follicle formation. They are subsequently transported to the vegetal pole cortex and undergo subsequent waves of vegetal or animal pole localization, where they persist and play essential roles in oocyte patterning and germ-cell formation in the early embryo. Some of the mRNAs initially deposited at the vegetal pole are later reoriented to define the future dorsal-ventral (d/v) axis (reviewed in Langdon and Mullins, 2011; Colozza and De Robertis, 2014; King et al., 2005). A similar sequence of events takes place in *Drosophila* oocytes on a greatly accelerated time scale, perhaps consistent with the much more rapid development of *Drosophila* oocytes generally. *Oskar* and *orb/CPEB* mRNAs move along the fusome and briefly associate with the Bb as it initially forms, before moving to the oocyte posterior (Cox and Spradling, 2003). Like in *Xenopus*, some originally posterior mRNAs such as *gurken* mRNA are later reoriented to establish the d/v axis, a process that in *Drosophila* occurs long after the Bb has dispersed and that involves further changes in the MT cytoskeleton, including new ncMTOCs (Milas and Telley, 2022).

Genetic analysis of Bb formation in zebrafish identified the *buckyball* gene, which does not affect initial Bb formation, but is essential for Bb persistence and for oocyte patterning (Marlow and Mullins, 2008; Elkouby and Mullins, 2017). Buckyball and Xvelo, its *Xenopus* ortholog (Claussen and Pieler, 2004; Boke et al., 2016), are predicted to be intrinsically disordered proteins (Brangwynne et al., 2009; Han et al., 2012; Seydoux, 2018). Formation of phase-separated compartments might stabilize the initial Bb, allowing it to persist for long periods and carry out its multiple functions in mRNA and germ granule localization.

## Mouse oocytes generate a highly conserved Bb-GV patterning axis, but its influence in the embryo has not been established

Whether the mammalian oocyte, like *Drosophila*, zebrafish, and *Xenopus* oocytes, contributes developmentally significant information to the early embryo has long been a question of interest (Beddington and Robertson, 1999; Gardner and Davies, 2003; Wu and Dean, 2020; Innocenti et al., 2022). The strong conservation in mouse oocytes of events associated with oocyte polarization, including development within a cyst, centrosome transfer from NCs, and centrosome assembly into a cytocentrum and Bb that defines a Bb-GV axis, suggests that these processes continue to play a significant role. Efforts to identify localized mRNAs arising in Bb-stage oocytes that move to the oocyte cortex have not been successful. Many challenges in comparing mammalian oocyte development to those in other groups are due to viviparity, which significantly delays embryonic development and patterning relative to non-eutherians, in favor of extraembryonic tissue growth needed to establish a maternal trophic connection (O’Farrell et al., 2004; Maltepe and Fisher, 2015). It is plausible that a new pathway evolved to transduce into the embryo the long-conserved patterning role for the polarized oocyte.

One clue may lie in the mammalian-specific genes that constitute the subcortical maternal complex (SCMC) (Wu and Dean, 2020) whose formation parallels that of the Bb. Depletion of SCMC components causes maternal infertility due to embryo arrest at cleavage stages (Li et al., 2010). One SCMC component, Floped, localizes to the apical part of the cell and cell cortex and is involved in establishing apical/basal polarity. Another Padi6 contributes to the cytoskeletal organization and is a component of ‘cytoplasmic lattices’ that form in the oocyte (Capco and McGaughey, 1986; Esposito et al., 2007). These structures persist in the early embryo and might transmit patterning information, although such a connection acting beyond the 2cell stage has not been demonstrated.

Another possibility is that the mammalian oocyte imprints embryonic pattern information onto a stable extracellular structure such as the zona pellucida (ZP) lying just outside the oocyte surface. The *Drosophila* oocyte carries out such a strategy to transduce the oocyte dorsal/ventral (d/v) axis to the fly embryo (review: Stein and Stevens, 2014; Merkle et al., 2020). Germ cell signals to the follicle cells cause them to alter the structure of the vitelline membrane that lies just outside the oocyte plasma membrane, with d/v asymmetry. These signals are later read out after fertilization by proteolysis, bind to oocyte surface receptors, and establish the d/v axis in the embryo (see: Nüsslein-Volhard, 2022).

Mouse extraembryonic endoderm (EE) represents a possible target since these cells plays a major role in patterning the inner cell mass by migrating anteriorly and forming the anterior visceral endoderm (AVE; reviewed in Stower and Srinivas, 2018). Gene expression in AVE cells establishes an a/p axis in the epiblast. It is currently not well understood what causes particular EE cells to migrate at an appropriate time and location to establish the AVE.

We propose that patterning information from the Balbinai body-containing early mouse oocyte is transferred locally to extracellular matrix (ECM) and forming zona pellucida. There is extensive evidence of secretory activity from the Bb-associated Golgi membranes of mouse oocytes at P4 (Lei and Spradling, 2016) and molecular evidence that granulosa cells synthesize ECM proteins at this time (Niu and Spradling, 2020a). It is plausible that asymmetries established in the ZP are read out in the embryo and program a limited number of precursor EE cells in the early embryo to become AVE precursors that establish the embryonic a/p axis. Regardless of exact mechanistic details, the ancient connection between the Bb-GV axis and the a/p axis may not have been lost at the time of eutherian evolution but modified to continue to provide its long-conserved role.

The story of how insights into the central roles of germline cysts were achieved contains a timely message. We would be ignorant today of the fundamental role of cysts in vertebrate and mammalian oogenesis had not studies of a wide range of organisms been supported and valued, especially of invertebrates and insects. Because evolution has conserved so much of animal biology, what matters in basic science is to study processes in systems that are favorable to advancing our understanding, while the particular species or group analyzed is intrinsically of less importance.

## Data Availability

Sequence data are available in the GEO database (GSE136441; Niu and Spradling, 2020b).
